# Polyhydroxylated Nanosized Graphite as Multifunctional Building Block for Polyurethanes

**DOI:** 10.3390/polym14061159

**Published:** 2022-03-14

**Authors:** Lucia Rubino, Giulio Torrisi, Luigi Brambilla, Luca Rubino, Marco Aldo Ortenzi, Maurizio Galimberti, Vincenzina Barbera

**Affiliations:** 1 Department of Chemistry, Materials and Chemical Engineering “G. Natta”, Politecnico di Milano, Via Mancinelli 7, 20131 Milano, Italy; luciarita.rubino@polimi.it (L.R.); giulio.torrisi@mail.polimi.it (G.T.); luigi.brambilla@polimi.it (L.B.); rubinoluca@live.it (L.R.); 2 Laboratory of Materials and Polymers (LaMPo), Department of Chemistry, Università degli Studi di Milano, Via Golgi 19, 20133 Milano, Italy; marco.ortenzi@unimi.it

**Keywords:** graphene layers, polymer, carbocatalysis

## Abstract

Polyurethane nanocomposites were prepared with a nanosized high surface area graphite (HSAG) functionalized on its edges with hydroxyl groups as a building block. Edge functionalization of HSAG was obtained through reaction with KOH. The addition of OH groups was demonstrated by means of infrared (FTIR) and thermogravimetric analysis (TGA), and the Boehm titration allowed estimation of a level of about 5.0 mmol_OH_/g_HSAG_. Results from wide-angle X-ray diffraction (WAXD) and Raman spectroscopy suggested that functionalization of the graphene layers occurred on the edges. The evaluation of the Hansen solubility parameters of G-OH revealed a substantial increase of *δ_P_* and *δ_H_* parameters with respect to HSAG. In line with these findings, homogeneous and stable dispersions of G-OH in a polyol were obtained. PU were prepared by mixing a dispersion of G-OH in *cis*-1,4-butenediol with hexamethylene diisocyanate. A model reaction between catechol, 1,4-butanediol, and hexamethylene diisocyanate demonstrated the reactivity of hydroxylated aromatic rings with isocyanate groups. PU-based G-OH, characterized with WAXD and differential scanning calorimetry (DSC), revealed lower T_g_, higher T_c_, T_m_, and crystallinity than PU without G-OH. These results could be due to the higher flexibility of the polymer chains, likely a consequence of the dilution of the urethane bonds by the carbon substrate. Hence, G-OH allowed the preparation of PU with a larger temperature range between T_g_ and T_m_, with potential positive impact on material applications. The model reaction between butylisocyanate and 1-butanol revealed that HSAG and G-OH promote efficient formation of the urethane bond, even in the absence of a catalyst. The effect of high surface area carbon on the nucleophilic oxygen attack to the isocyanate group can be hypothesized. The results here reported lead us to comment that a reactive nanosized sp^2^ carbon allotrope, such as G-OH, can be used as a multifunctional building block of PU. Indeed, G-OH is a comonomer of PU, a promoter of the polymerization reaction, and can definitely act as reinforcing filler by tuning its amount in the final nanocomposite leading to highly versatile materials. The larger temperature range between T_g_ and T_m_, together with the presence of G-OH acting as a reinforcing agent, could allow the production of piezoresistive sensing, shape-memory PU with good mechanical features.

## 1. Introduction

Sp^2^ carbon allotropes play a key role in material science. Carbon black (CB) has been used for over a century as a reinforcing filler and is one of the top ten most important chemical substances [1,2]. It is composed of primary nanoparticles that form micron-sized aggregates. Over the last decades, after the discovery of fullerene [3,4], nanosized carbon allotropes such as carbon nanotubes (CNT) [5,6,7,8], graphene (G), and graphene-related materials GRM [9,10,11,12,13] have exponentially increased their importance [5,6,7], also as ingredients of polymer composites. They can be separated into individual nanometric particles and are able to establish a large interfacial area with the polymer matrix, with significant impact on its material properties. Nanosized carbon allotropes aggregates can be broken down into primary particles, which are characterized by a high surface area and hence a large polymer–filler interfacial area, improving the properties of the material.

Indeed, thanks to the nano-fillers, polymer composites achieve high performance, even when they are based on so-called commodity polymers. Polymer composites obtained using nano carbon allotropes find widespread applications in fields such as automotive, aeronautics, building and construction, as well as energy, and in the chemical industry in general. The main factors which steer their properties are: (I) the distribution and dispersion of the filler(s) in the polymer matrix; (II) the interaction between the polymer and the filler.

In the realm of polymers, polyurethanes (PU) are probably the most versatile, as they can be thermoplastic, elastomeric, thermoset, and foams [14]. PU are step-growth polymers, commonly synthesized by means of a polyaddition reaction between isocyanates, polyols, and chain extenders in the presence of a catalyst to accelerate the polymerization reaction [15,16,17]. The applications of PU range from structural materials to foam padding, insulation in buildings and fridges, parts in cars, furniture and beds, shoe soles, coating, and adhesives [18]. Thus, it is not surprising that PU represent one of the most important polymer families, since their production is estimated to be more than 18 million tons per year, about 5 mass % of the total worldwide polymer production [19,20]. Among the polyurethane-based composites [21], those containing carbon materials are investigated for their mechanical, thermal, electrical, and piezoresistive properties [22], and many reports are available in scientific literature on PU composites based on CB, CNT, and GRM [23,24,25,26,27,28,29,30,31,32,33,34,35,36].

PU composites with GRM [28,29,30,33,34,35,36] were prepared for improving mechanical [28,30,35], thermal and flame [28,33,34,35], and electrical [29,34] properties, and for preparing materials for filtering [36]. Expanded graphite promoted the enhancement of the thermal stability and the flame retardancy in castor oil phosphate-based rigid polyurethane foam [33], of the mechanical and fire properties in crude glycerol polyurethane foams [35], as well as of electrical and thermal conductivities in foams obtained from renewable resources [34]. Moreover, it was combined with waterborne polyurethane, developing porous foams with underwater oleophobic properties [36]. The compatibilization of the graphitic material with the PU matrix was achieved by using either graphene oxide, obtained by means of the Hummers method [37], subsequently performing the in situ polymerization [28], or reduced graphene oxide (Hummers method to GO followed by thermal reduction at 1050 °C) with a solution blending [29]. A single layer graphene, without chemical treatments, was mixed with PU through solution blending, melt blending (cryo-grinding of PU), and in situ polymerization (blending and degassing G with a PU pre-polymer). The procedures adopted for the preparation of GRM/PU composites appear hardly scalable and, in particular, covalent bonding is achieved by performing the oxidation of graphite, a reaction which is characterized by harsh conditions and noxious reagents. It would indeed be highly desirable to identify a versatile method, suitable to the functionalization of GRM and to the promotion of their integration in a PU matrix thanks to a chemical bond between the graphitic material and the PU matrix itself, allowing preparation of the PU/GRM composites through a simple and scalable technique. It would be even more desirable if such a method could be applied to all the families of sp^2^ carbon allotropes in order to obtain PU nanocomposites which are highly versatile and have intriguing features, due to an efficient dispersion of graphene, which could allow the pursuit of frontier applications. In particular, target applications for the PU/GRM nanocomposites could be in piezoresistive sensors [38,39,40,41,42] by using hybrid systems with CNT [41] and with silica nanoparticles to achieve the self-healing ability of the materials [42]. The polar groups on the carbon surface would indeed be beneficial. Moreover, PU/GRM nanocomposites could be used for shape memory materials [43,44].

The research reported here was inspired by these objectives. Polyurethanes were prepared in the presence of a polyhydroxylated nanosized graphite (G-OH). The hydroxyl functional group was selected in order to establish a chemical bond between the PU chains and the carbon allotrope: G-OH was thus conceived as a building block of PU and focus was put on the study of PU preparation with G-OH as a comonomer.

The selected pristine nanosized graphite had high surface area and high shape anisotropy (HSAG) [45], which means a high crystalline order inside the plane and a low number of layers stacked, in crystalline domains, in the orthogonal direction. In previous studies [46,47], it was shown that graphene layers can be functionalized with hydroxyl groups in peripheral positions, essentially on the edges, by performing their reaction with KOH, obtaining polyhydroxylated graphene layers with a substantially unaltered bulk structure. An aromatic nucleophilic substitution by KOH on the edge of the graphene layers was hypothesized. The polyhydroxylated layers were the substrate for further chemical reactions [48], such as the Reimer Tiemann and the Cannizzaro reactions, with the selective introduction of aldehydic and acidic functional groups on the edges of the layers. The reactivity of HSAG edges was also demonstrated by performing other functionalization reactions [49,50].

In this work, the edge functionalization of graphene layers with OH functional groups were performed through the reaction of HSAG with KOH [46,48], preparing the product called G-OH.

The introduction of edge OH groups on graphene layers could also be obtained through the oxidation of the graphitic substrate with hydrogen peroxide [51,52]. However, it has been shown [52] that the oxidation of graphitic substrates with H_2_O_2_ can lead to a variety of oxygenated functional groups, as it is very sensitive to experimental conditions. Pristine and functionalized graphene layers were characterized by means of wide-angle X-ray diffraction (WAXD), Fourier Transformed Infrared spectroscopy (FTIR), and Raman spectroscopy. The Hansen Solubility Parameters (HSP) and Hansen Solubility Sphere (HSP) of G-OH were also determined by studying the behavior of G-OH dispersions in different solvents. To investigate the reactivity of the hydroxylated nanographite with an isocyanate, a model reaction was performed by using 1,2-cathecol as a model system. The product was analyzed by means of Fourier Transform Infrared spectroscopy and ^1^H and ^13^C NMR.

**Figure 1:**
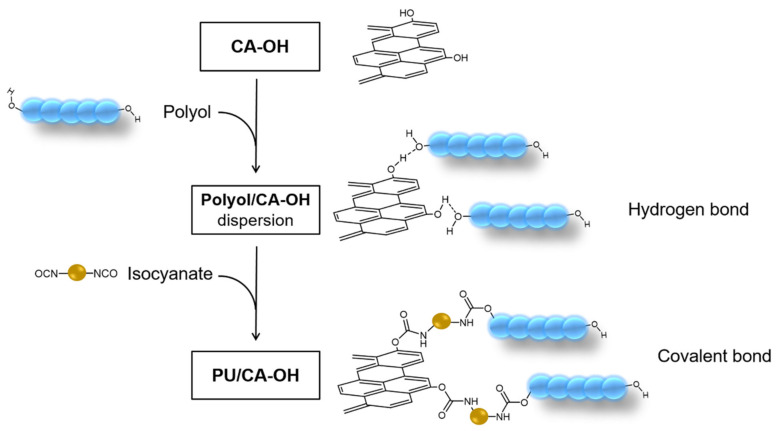
Scheme for the in situ preparation of polyurethane in the presence of sp^2^ carbon allotropes.

The scheme for the preparation of a PU based on G-OH as the building block is shown in Figure 1.

PU composites were prepared by mixing the graphitic material, either pristine or functionalized with a diol, then promoting polymerization by adding hexamethylene diisocyanate (HDI) (Figure 1). The crystalline structure of the composites was investigated by means of wide-angle X-ray diffraction (WAXD). Thermal properties were studied by means of differential scanning calorimetry (DSC). The catalytic effect of the nanographite on the nucleophilic oxygen addition to the isocyanate group was investigated by performing the model reaction between butylisocyanate and 1-butanol.

## 2. Experimental

### 2.1. Materials

#### 2.1.1. Carbon Allotropes

High surface area graphite (HSAG) was Nano24 (Asbury Graphite Mills Inc., New Jersey, USA), with carbon content reported in the technical data sheet of at least 99 wt%. Chemical composition determined from elemental analysis was, as wt%: carbon 99.5, hydrogen 0.4, nitrogen 0.1, oxygen < 0.05. BET surface area was 330 m^2^/g and DBP absorption was 162 mL/100 g [50]. Preparation and characterization of G-OH have been performed as reported in [46]. Details are in Supplementary Materials (Sections S1 and S2).

#### 2.1.2. Chemicals

Reagents and solvents commercially available were purchased and used without further purification: Chemical for functionalization. Potassium hydroxide pellets (Carlo Erba Reagenti, Cornaredo, Italy).

Chemicals for the determination of solubility parameters and for the dispersion in polyol. Ethanol, glycol, 2-propanol, acetone, ethyl acetate, chloroform, xylene, toluene, and hexane (Sigma-Aldrich, Milan, Italy) (Reagent grade). Polyether polyol was VORANOL 1010L with OH index of 115 mg KOH g^−1^ (DOW Chemical, Michigan, USA).

Chemicals for the polymerizations. Diol was *cis*-1,4-butendiol (Sigma-Aldrich, Milan, Italy) (reagent grade). The isocyanate was hexamethylene diisocyanate (Fluka, reagent grade). The catalyst was 1,4-diazabicyclo[2.2.2]octane (DABCO) (Sigma-Aldrich, Milan Italy) (reagent grade).

Chemicals for model reactions. 1,4-butanediol, cathecol, 1,4-diazabicyclo[2.2.2]octane (DABCO).

### 2.2. Preparation of the Dispersions and Stability Evaluation

A polyol and either HSAG or G-OH were poured in a flask. Dispersions were prepared with different concentrations: 0.5, 0.1, 0.05, 0.01, 0.005, 0.001 mg/mL. The mixture was sonicated for 15 min using a 2 L ultrasonic bath.

UV-Vis absorption was measured for each suspension after sonication, after 3 and 7 days, and after centrifugation at 3000 and 6000 rpm for 30 min.

### 2.3. Synthesis of Polyurethane

In a 100 mL beaker equipped with a magnetic stirrer, the diol, the catalyst DABCO, and HDI were poured in sequence. The mixture was mechanically stirred until the solid was formed. When used, HSAG or G-OH were added to the diol. Formulations, recipes, and reaction conditions are in Table 1.

### 2.4. Characterization of Polyurethane

PU were characterized by means of WAXD and DSC (see Section S2).

### 2.5. Model Reactions

#### 2.5.1. Model Reaction for the Synthesis of PU

Catechol (3.12 g, 28.3 mmol), 1,4-butanediol (2.55 g, 28.3 mmol), and DABCO (0.15 g, 1.34 mmol) were mixed in a 100 mL flask. After few minutes, HDI (9.53 g, 56.7 mmol) was added and the mixture was left stirring at room temperature for 5 min. 645 [M+H]^+^.

NMR analysis of the reaction product.

^1^H-NMR and ^13^C-NMR spectra were recorded on a Bruker 400 MHz (100 MHz ^13^C) instrument at 298 K. Chemical shifts were reported in ppm with the solvent residual peak as internal standard (DMSO-*d*_6_: *δ*_H_ = 2.50 ppm, CDCl_3_: *δ*_H_ = 7.26 ppm).

^1^H NMR (400 MHz, DMSO-d6, δ in ppm): 1.38 (m, 20H); 1.56 (s, 4H); 3.02 (m, 10H); 3.39 (t, 1H); 3.93 (s, 4H); 7.01 (m, 2H), 7.17 (m, 4H), 7.70 (m, 2H).

^13^C NMR (400 MHz, DMSO-d6, δ in ppm): 25.9, 26.4, 29.4, 29.6, 30.0, 47.3, 60.8, 63.6, 117.0, 119.3, 124.3, 126.0, 143.9, 154.1, 156.7.

#### 2.5.2. Model Reaction for Studying the Effect of G and G-OH on the Formation of Urethane Bond

Butanol (0.7 g, 9.8 mmol) and butylisocyanate (0.9 g, 9.8 mmol) were mixed in a 50 mL round bottom flask at room temperature. After 1, 2, and 12 h, reaction was checked by means of GC-MS.

The same reaction was performed by adding 0.03% mol of G or G-OH to butanol (moles of benzene rings were considered). The reaction products were investigated by means of FTIR.

## 3. Results and Discussion

### 3.1. Functionalization of Carbon Allotropes

Functionalization of HSAG with hydroxyl groups was obtained by performing the reaction of HSAG with KOH. First experiments performed with HSAG as the carbon allotrope have already been reported [46]. The procedure used in the present research has already been published [46,49]. In brief, KOH and the carbon allotrope were mixed with a KOH/G mass ratio = 1:5 and were heated at 100 °C for 3 h in the absence of solvents.

The number of functional groups introduced in HSAG was estimated by means of Boehm titration [52] (see Section S2), a method able to detect the number of acidic groups, and a value of 5.0 mmol/g was found. Such relatively high value could be ascribed to the large surface area of HSAG (330 m^2^/g, from nitrogen absorption experiment). However, a value of 3 mmol/g was found for multiwalled CNT [52], which had a similar surface area (275 m^2^/g). Taking into consideration the mechanism hypothesized for the functionalization with KOH (the nucleophilic substitution), the large number of edges available in the nanographite appears to be responsible for the higher reactivity.

Pristine G and G-OH samples were characterized by means of FTIR and Raman spectroscopies. Infrared spectra of pristine G and G-OH are reported in Figure S1. Functional groups in G-OH were detected by analyzing the samples in a Diamond Anvil Cell (DAC). All the spectra in Figure S2 are characterized by an increasing background toward high wavenumbers due to diffusion/reflection phenomena of the IR light by the particles of the sample. The assignment of peaks is reported as Supplementary Materials (Section S3).

Raman spectroscopy is widely employed for the study of carbonaceous materials [53,54,55,56,57,58,59,60,61,62,63]. The Raman spectra of HSAG and G-OH (recorded with the laser excitation at 632.8 nm) are reported in Figure S2. By comparing the spectra of pristine HSAG with that of G-OH, no indication was found that the reaction with KOH appreciably alters the structure of the starting graphitic material.

### 3.2. Calculation of the Hansen Solubility Sphere and Hansen Solubility Parameters

It has been reported that close values of the solubility parameters of a carbon nanomaterial and a polymer is the prerequisite to achieve even distribution and dispersion of the nanomaterial in the polymer matrix [46,64,65,66,67,68] with its separation into individual particles [66,69]. The Hildebrand and Hansen solubility parameters (HSP) of G-OH adducts were evaluated [64,65]. The basic concepts of the solubility parameters are discussed as Supplementary Materials, with indication to the relevant scientific literature. In brief, the Hansen method takes into account the molecular interactions between a solute and a solvent and allows the evaluation of solubility parameters based on the following interactions: *δ_D_* (dispersion), *δ_P_* (polar), *δ_H_* (hydrogen bonding). The Hildebrand (total) *δ_T_* solubility parameter is calculated as the sum of the squares of the HSP. Details are reported in Supplementary Materials (Section S4).

Dispersions of G-OH were prepared in ten different solvents, with solubility parameters *δ_T_* in the range from 30 (water) to 15 (hexane). The stability of the dispersions was qualitatively investigated, as described in the Experimental section. In brief, dispersions were sonicated for 30 min and visual inspection was made after one hour and one week, classifying them as “good” or “bad”, in case a homogenous dispersion or a separation of the black powder were observed, respectively. It is worth commenting that “bad” dispersions were observed even after only one hour, whereas “good” dispersions were stable after one week. The results of the visual inspection are in Table S1 in the Supplementary Materials. Estimation of the HSP *δ_H_* (hydrogen bonding), *δ_D_* (dispersion), *δ_P_* (polar), and *δ_T_* (total) for the G-OH adducts was made on the basis of the data of Table S1 and applying the algorithm described in Figure S3. Solubility spheres, which include the good solvents and exclude the bad ones, were generated. Values of the HSP and of the radius of the Hansen sphere are shown in Table 2, for both G-OH and the pristine allotrope.

**Figure 2:**
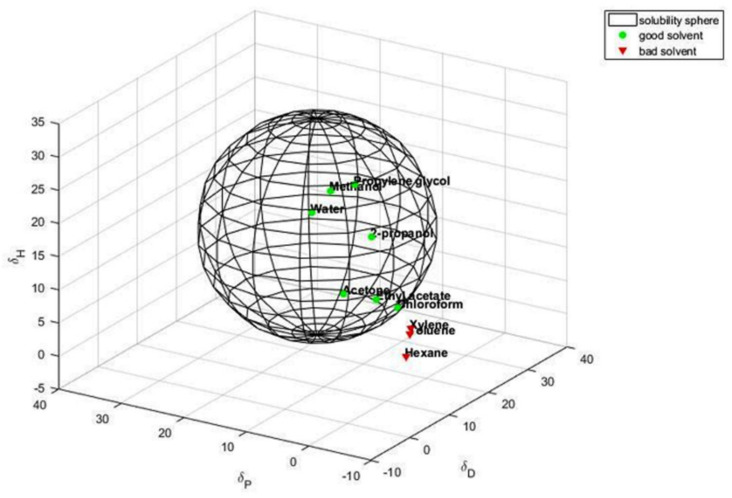
Hansen solubility sphere calculated for G-OH. The green circles correspond to the good solvents (within the radius of interaction), the red triangles to the bad solvents (outside the sphere).

The sphere for GOH is shown in Figure 2.

Polar protic and aprotic solvents such as water, 2-propanol, and acetone are included in the G-OH interaction sphere, whereas the apolar solvents are outside of the sphere. With respect to HSAG, G-OH shows higher values of *δ_D_* and *δ_H_*.

On the basis of these findings, it can be reasonably assumed that an even dispersion of G-OH can be obtained in a diol. Moreover, as discussed in the Introduction, the OH group is expected to react with isocyanate during the preparation of PU.

### 3.3. Dispersion of HSAG and G-OH in Polyol

Dispersions of G-OH in a polyol were studied, particularly in view of the scale up of the PU preparation discussed in the present work. Indeed, homogenous and stable dispersion of a nanosized graphite in polyol would be necessary at pre-industrial scale. Dispersions were prepared at different concentrations (1, 0.1, 0.05, 0.01, 0.005, and 0.001 mg/mL), as described in the experimental part. Figure S4 shows dispersions of G-OH in a range of concentration from 0.1, to 0.001 mg/mL and at 1 mg/mL as the concentration, at rest, after 1 month of storage. Figure S5 reports results from UV-Vis absorption analysis. Figure S5A shows the absorbance detected for the freshly prepared G-OH polyol suspensions (1 mg/mL). Figure S5B shows that the absorbance monotonously increases with G-OH concentration.

### 3.4. Reactivity of Polyhydroxylated Carbon Allotropes with Isocyanates: Model Reaction

**Figure 3:**
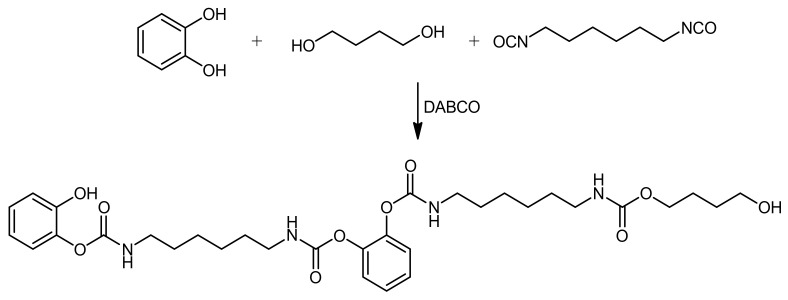
Reaction between 1,2-catechol, 1,4-butanediol, hexamethylene diisocyanate (DABCO as the catalyst).

A model reaction was performed to investigate the reactivity of hydroxylated aromatic rings, such as those in G-OH, with an isocyanate in the presence of a traditional chain extender for PU, such as 1,4-butanediol. 1,2-catechol was selected as the model aromatic compound. The pathway of the model reaction is shown in Figure 3.

**Figure 4:**
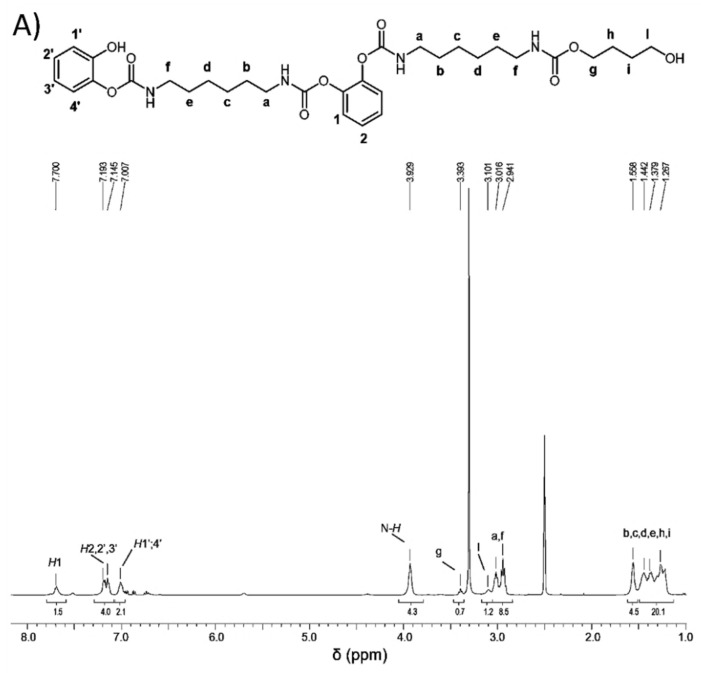

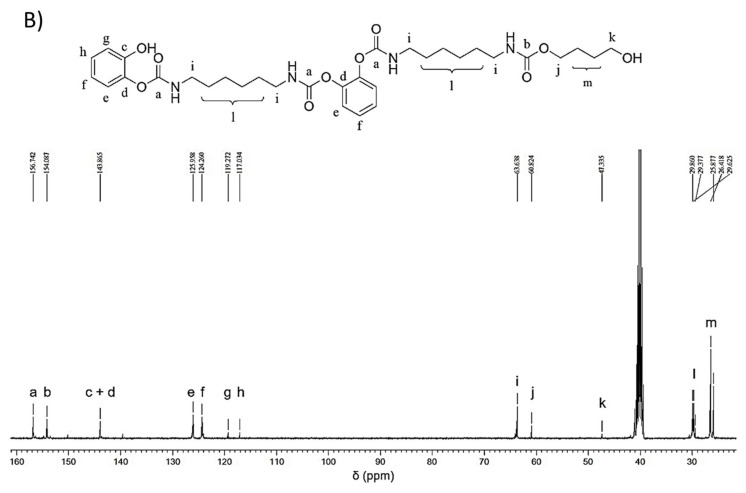
(**A**) ^1^H (400 MHz) and (**B**) ^13^C NMR (100 MHz) spectra in DMSO-*d*_6_ of PU obtained by reacting 1,2-catechol, 1,4-butanediol, and hexamethylene diisocyanate.

The reaction was performed at room temperature by mixing 1,2-catechol, 1,4-butanediol, HDI, and DABCO as the catalyst. The reaction product, the oligourethane, was analyzed by means of ^1^H-NMR, ^13^C-NMR, and FTIR spectroscopies, and the spectra are shown in Figure 4A,B and Figure S6, respectively. As it is shown in Figure 4A,B, the assignment of the peaks in the spectra is consistent with the structure hypothesized for the product. Signals due to the monomers cannot be detected.

The number average molecular mass (${\bar{M}}_{n}$) of the oligo-urethane was calculated through the ratio between the intensities in the ^1^H-NMR spectrum of the chain end CH_2_-OH signal (l) (at 3.1 ppm) and of the signal due to the (N-H) urethane linkage (at 3.8 ppm), and was estimated to be equal to 646 g/mol.

In the FTIR spectrum, peaks due to the monomers cannot be detected, whereas it is possible to observe two bands related to chemically different urethane linkage: C=O stretching of -CH_2_-O-CO-N at 1700 and C=O stretching of Ph-O-CO-N at 1710 cm^−1^. In the ^13^C NMR spectra, the carbonyl groups are at 154 and 156 ppm for the alkyl and aryl urethanes, respectively.

### 3.5. Polyurethane Composites

A solventless synthesis of PU was carried out by simply dispersing G-OH in the diol, then mixing this dispersion with hexamethylene diisocyanate and the catalyst, obtaining a fast polymerization (in about 10 s). The scheme for the preparation of the composite material is shown in Figure 1. Recipes and polymerization conditions are in Table 1.

The PU/G-OH composites were studied by means of FTIR, WAXD, and DSC.

**Figure 5:**
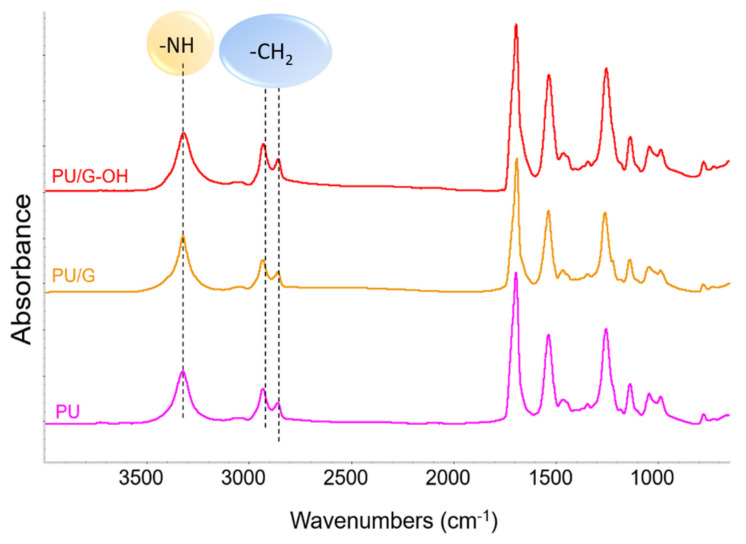
FTIR spectra of PU, PU/G, and PU/G-OH.

The nature of functional groups was investigated by means of FTIR spectroscopy. Figure 5 shows the FTIR spectra of the reaction products from Runs 1 (PU), 2 (PU/G), and 3 (PU/G-OH) of Table 1.

The patterns are compatible with those of a polyurethane. In the high wavenumber region, the following can be observed: (i) the strong peak of NH stretching vibration at 3326 cm^−1^ (indicated by a dotted line in the figure), (ii) the broad and weak absorption in the range 3100–3000 cm^−1^ assigned to aromatic CH stretching, and (iii) the symmetric and asymmetric stretching vibrations of CH_2_ groups at 2934 and 2860 cm^−1^. The other main peaks were assigned as follows: to C=O stretching at 1694 cm^−1^, to NH deformations at 1536 cm^−1^, to CH_2_ bending, at 1460 cm^−1^, and to CO stretching at 1255cm^−1^. The FTIR spectra show similar features. Taking the signal due to CH_2_ stretching as reference, a lower relative intensity for the NH stretching in the spectrum of PU/G-OH can be observed. This suggests that G-OH acts as comonomer in the PU chain. These results appear to be in line with what is reported in the literature [70,71,72,73]. GO with oxygenated functional groups on the basal planes of the graphitic layers was added in a low amount (0.04 wt%) to waterborne polyurethane urea dispersions, synthesized by using polyadipate of 1,4-butanediol polyol and isophorone diisocyanate, which led to some differences in the intensities of the NH stretching band and to the displacement of the NH band to a lower wavenumber. These findings were attributed to the reaction of the isocyanate groups with the oxygenated functional groups of the carbon filler.

**Figure 6:**
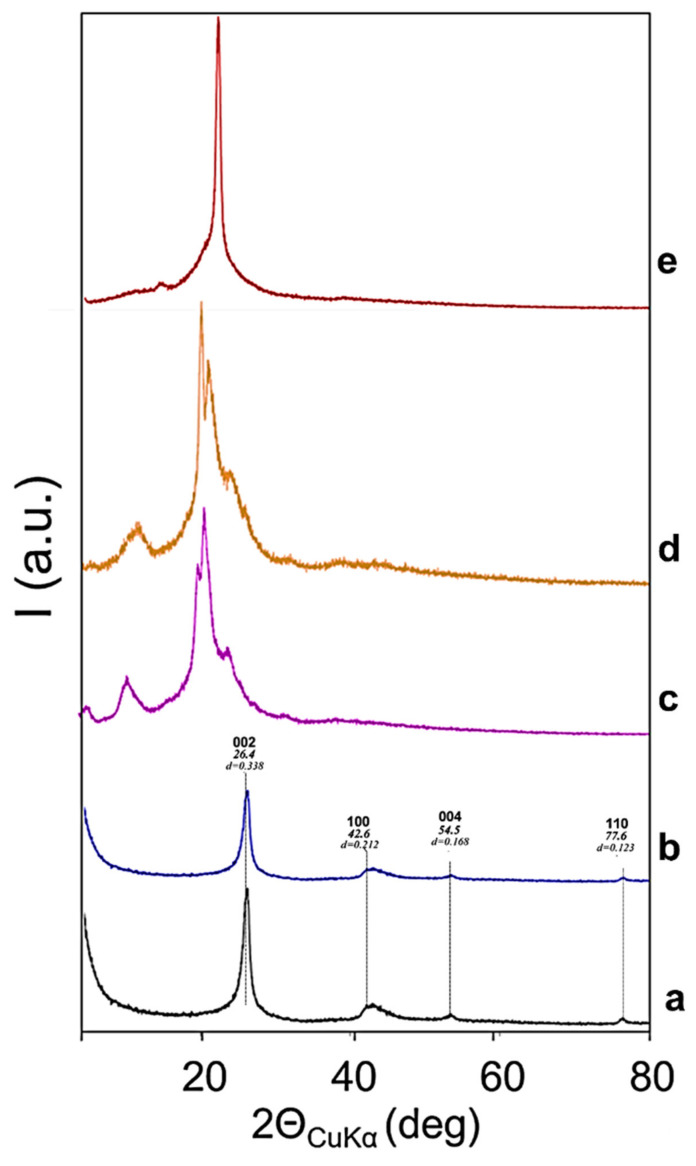
WAXD patterns of: HSAG (**a**), G-OH (b), PU (**c**), PU/G (**d**), and PU/G-OH (**e**).

The organization at the solid state was studied by means of WAXD. Figure 6 shows WAXD patterns taken on powders of HSAG, G-OH, PU, PU/G, and PU/G-OH.

The number of stacked layers in HSAG and G-OH samples was calculated by applying the Scherrer equation to 002 reflection (see Section S6).

The X-ray diffraction patterns of PU and the PU nanocomposites with G and G-OH are shown in Figure 6c–e, respectively. The PU pattern reveals four broad crystalline peaks centered at 5.5, 10.8, 20.4, 21.1, and 24.2 as the 2Θ values. A similar pattern is shown by PU/G composite. The incorporation of G-OH seems to increase the crystallinity of PU: a clear crystalline peak can be observed at 22.2 (2θ). It is interesting that the incorporation of G-OH induces the formation of a different crystalline structure, whereas pristine HSAG, which does not react with isocyanate, does not. In the above-mentioned literature [70,71,72,73], an increase of the intensity of the PU X-ray diffraction peaks, even by adding a low amount of GO (0.04 wt%), was reported.

DSC heating scans were taken on PU, PU/G, and PU/G-OH composites. Table 3 shows data collected from the thermal analysis. T_g_, T_m_, and ΔH_m_ refers to the second heating scan.

All the samples revealed a visible T_g_, where value decreases in the presence of G and, in particular, of G-OH, to indicate a higher mobility of the polymer chains. It is known that the rigidity of PU is due to the urethane bonds, in particular to their supramolecular interactions. It could be commented that the carbon filler G dilutes the urethane bonds in the composite matrix. The decrease of T_g_ value in the presence of G-OH could seem surprising, as G-OH acts as a crosslinker of the PU chains. It could be hypothesized that the formation of the urethane linkage on G-OH surface prevents the supramolecular interactions of the PU chains. In the above-mentioned literature [70,71,72,73], the influence of the addition of a reactive graphitic filler, such as GO, though in a low amount 0.04% wt, was documented. Tg was reported either to decrease [70] or to increase [72], however in a very low temperature range and a melting peak appeared in the DSC trace [73]. The effect of the graphitic filler appears to depend on the chemical structure of PU, as well as on the composite preparation procedure.

As shown by WAXD analyses, all PU are semi-crystalline and are thus characterized by T_c_ and T_m_, where values increase in presence of G and, in particular, of G-OH, the latter leading to an increase of both T_c_ and T_m_ of about 10 °C. Additionally, the value of ΔH_m_ appears to be higher, though only slightly, in the case of the composite with G-OH. These results are in line with WAXD findings, which indicated the enhancement of PU crystallinity in presence of G-OH. The higher mobility of the polymer chains can account for an easier crystallization of the nanocomposite.

It is indeed worth observing that G-OH allows the preparation of PU materials with larger operating windows.

**Figure 7:**
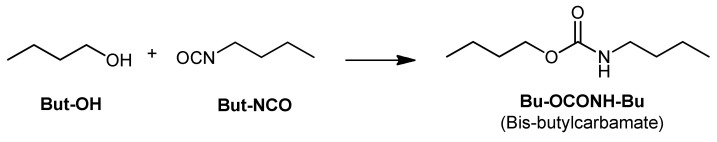
Reaction between butylisocyanate and 1-butanol.

The findings commented so far appear to confirm that G-OH is a building block of PU. It could also be hypothesized that the sp^2^ carbon allotrope could have a catalytic effect on the nucleophilic oxygen addition reaction. It is well known that both graphene and graphite oxide show catalytic effects on many organic reactions, being more effective and selective than other catalysts [48]. The effect of the carbocatalysis on the formation of amides has been reported, [74] as well as the effect of phenolic resins [75,76]. The possible catalytic activity of G and G-OH on reactions between monofunctional low molecular mass isocyanate and alcohol, such as butylisocyanate (But-NCO) and 1-butanol (But-OH), was studied. The scheme of the reaction is in Figure 7.

The reactions were carried out at room temperature under solvent free conditions, with or without G and G-OH, used at 3% by mass level, as in the case of the above-discussed PU. Checking the reaction was done by means of ^1^H-NMR. Reaction conditions and yields are in Table 4.

The carbon filler led to a higher yield of the reaction in a much lower time. The slightly lower yield detected in the presence of G-OH was hypothesized to be due to the reactivity of G-OH with the isocyanate. In order to demonstrate this reactivity, G-OH, used in the reaction reported in Run 3 of Table 4, was isolated and analyzed by means of FTIR spectroscopy. The FTIR spectrum, reported in Figure S7, reveals signals due to the carbamate linkage: NH stretching vibration and C=O stretching at 3326 cm^−1^ and at 1688 cm^−1^, respectively. Signals which can be attributed to the alkyl chain are visible at 2870 and 2950 cm^−1^ for CH_2_ and CH_3_, respectively.

These findings suggest that the nanosized graphite plays a role in promoting the nucleophilic addition of oxygen to the isocyanate group. This would mean that a nanosized graphite, functionalized with a hydroxyl group, could have a manifold function in a PU matrix as a promoter of the reaction and building block for the formation of PU and as a reinforcing filler, by tuning its amount in the final nanocomposite.

## 4. Conclusions

This work reveals that it is possible to prepare polyurethanes nanocomposites with a nanosized high surface area graphite functionalized with hydroxyl groups as building blocks, i.e., as a comonomer for PU.

The results reported above show that the functionalization of a high surface area graphite with KOH is a robust and reproducible reaction, which leads to the introduction of a controlled amount of OH groups. Characterization techniques such as IR, TGA, and Boehm titration allowed investigation of the type and the amount of OH groups, which was found at a level of about 5 mmol/g_HSAG_. WAXD and Raman analyses clearly suggested that the functionalization occurred on the edges of HSAG. Homogeneous dispersions of G-OH in a polyol were prepared, in line with the evaluated Hansen solubility parameters, which revealed the increased polarity of HSAG.

G-OH was used as a comonomer for PU synthesis by simply preparing a dispersion in 1,4-butanediol, then performing the reaction with hexamethylene diisocyanate. A successful model reaction, between catechol and 1,4-butanediol, supported the formation of a urethane bond anchored on the carbon surface.

PU based on HSAG and, in particular, G-OH, revealed lower T_g_, higher T_c_, T_m_, and crystallinity: PU containing G-OH revealed an increase of 15 °C in the T_g_-T_m_ range in comparison to PU, also showing an increase of 10 °C in T_c_, confirming a higher tendency to crystallize. The dilution of the urethane linkages, by the carbon material, seems to promote a larger flexibility of the polymer chains and thus an easier crystallization. G-OH appears to lead to a wider PU operating window.

The experimental conditions used in the research here reported appear to be suitable for a pre-industrial scale upwards. Oxidation of the nanographite with KOH was selected, as it allows the specific introduction of OH groups on the edges of the graphene layers. However, other functionalization reactions, such as that with H_2_O_2_, can also be taken into consideration. More interestingly, G-OH appears to have manifold functions: as a promoter of polymerization reaction, as a comonomer, and definitely as a reinforcing filler, by tuning its amount in the final composite.

This work appears to pave the way for a new family of PU nanocomposites, based on functionalized nano sp^2^ carbon allotropes, the reactivity of which allows their integration in the composite matrix.

## Figures and Tables

**Table 1:** Synthesis of polyurethanes: formulations, recipes and reaction conditions.

Run	PU Sample	Diol ^a^	Isocyanate ^b^	Catalyst ^c^	Filler
		[g]	[g]	[mg]	(3 %w)
1	PU	10	19	2.54	n.u. ^d^
2	PU/G	10	19	2.54	HSAG
3	PU/G-OH	9.42 ^e^	19	2.54	G-OH

^a^*cis*-1,4-butenediol; ^b^ hexamethylene diisocyanate; ^c^ DABCO; ^d^ not used; ^e^ the number of OH groups was corrected taking into consideration the OH groups of G-OH.

**Table 2:** Hansen solubility parameters and sphere radius for pristine G and G-OH adducts ^a^.

Sample	*δ_D_*	*δ_P_*	*δ_H_*	Radius	*δ_T_* ^b^	Ref
G	18	2	4	1	22	49
G-OH	16	15	16	16	27	This work

^a^ Measure unit: MPa^1/2^; ^b^ *δ_T_*^2^ = *δ_D_*^2^ + *δ*_P_^2^ + *δ_H_*^2^.

**Table 3:** T_g_, T_m_, and ΔH_m_ for composites of Table 1 ^1^.

Sample	T_g_ (°C)	T_c_ (°C)	ΔH_c_ (J/g)	T_m_ (°C)	ΔH_m_ (J/g)
PU	12	88	34	121	34
PU/G	10	87	28	126	28
PU/G-OH	8	99	34	130	37

^1^ obtained via DSC.

**Table 4:** Reaction between butylisocyanate (But-NCO) and butanol (But-OH): reaction conditions, yields.

Run	Catalyst (3 %w) ^a^	Time (h)	Yield (%) ^b^
1	=	24	84
2	HSAG	2	93
3	G-OH	2	88

^a^ Molar ratio But-OH:But-NCO = 1:1; ^b^ All the yields refer to isolated chromatographically pure compounds, whose structures were confirmed by analytical and spectroscopic data.

## Supplementary Materials

The following supporting information can be downloaded at: https://www.mdpi.com/article/10.3390/polym14061159/s1, Section S1. Synthesis of G-OH: reaction of HSAG with KOH; Section S2. Characterization of HSAG, G-OH and PU; Section S3. FT-IR investigation on sp2 carbon allotropes; Figure S1. (A) FT-IR spectra of HSAG; (B) FT-IR spectra of G-OH.; Figure S2. Raman spectra with normalized intensities of HSAG (a), G-OH (b); Section S4. Calculation of the Hansen Solubility Parameters and Hansen Solubility Sphere; Figure S3. MATLAB algorithm used for the preparation of Hansen’s sphere; Table S1. Hansen solubility parameters for selected solvents and results of inspection of dispersions of GOH adducts after 1 week; Section S5. Polyol dispersions of G-OH; Figure S4. Polyol suspensions of G-OH (1 mg/mL) at rest (a) and at concentration of 0.1, 0.05, 0.01, 0.005 and 0.001 mg/mL (b) (from right to left); Figure S5. (A) UV-Vis traces of polyol dispersions of G-OH. (B) G-OH dispersions at different concentrations (1, 0.1, 0.05, 0.01, 0.005, 0.001 mg/mL).; Section S6. WAXD; Figure S6. FT-IR spectrum of PU from 1,2-catechol, 1,4-butanediol and hexamethylene diisocyanate; Figure S7. FT-IR spectrum of the reaction of 1-butanol with butylisocyanate and G-OH.
