# Supplementary material for: Polyhydroxylated Nanosized Graphite as Multifunctional Building Block for Polyurethanes
Source: Polymers (Basel). 2022 Mar 14;14(6):1159. doi: 10.3390/polym14061159 (PMC8953097; doi:10.3390/polym14061159)
Supplement: Supplementary file 1 [file polymers-14-01159-s001.zip › polymers-1473796-supplementary.pdf]

polymers

MDPI

Article

Polyhydroxylated Nanosized Graphite as Multifunctional Building Block for Polyurethanes

Lucia Rubino [1,†], Giulio Torrisi [1,†], Luigi Brambilla [1], Luca Rubino [1], Marco Aldo Ortenzi [2], Maurizio Galimberti [1,*] and Vincenzina Barbera [1,*]

[1] Politecnico di Milano, Department of Chemistry, Materials and Chemical Engineering "G. Natta", Via Mancinelli 7, 20131 Milano, Italy; luciarita.rubino@polimi.it (L.R.); giulio.torrisi@mail.polimi.it (G.T.); luigi.brambilla@polimi.it (L.B.); rubinoluca@live.it (L.Ru.)
[2] Laboratory of Materials and Polymers (LaMPo), Department of Chemistry, Università degli Studi di Milano, Via Golgi 19, 20133 Milano, Italy; marco.ortenzi@unimi.it
* Correspondence to: maurizio.galimberti@polimi.it (M.G.); vincenzina.barbera@polimi.it (V.B.)
† These authors contributed equally to this work.

Citation: Rubino, L.; Torrisi, G.; Brambilla, L.; Rubino, L.; Ortenzi, M.A.; Galimberti, M.; Barbera, V. Polyhydroxylated Nanosized Graphite as Multifunctional Building Block for Polyurethanes. *Polymers* **2022**, *14*, 1159. https://doi.org/10.3390/polym14061159

Academic Editor(s): José Miguel Martín Martínez

Received: 18 January 2022
Accepted: 8 March 2022
Published: 14 March 2022

Publisher's Note: MDPI stays neutral with regard to jurisdictional claims in published maps and institutional affiliations.

Copyright: © 2022 by the authors. Licensee MDPI, Basel, Switzerland. This article is an open access article distributed under the terms and conditions of the Creative Commons Attribution (CC BY) license (https://creativecommons.org/licenses/by/4.0/).

Supplementary Material

S1. Synthesis of G-OH: reaction of HSAG with KOH

In a 500 mL round bottom flask equipped with a magnetic stirrer HSAG (10 g, 139 mmol), KOH powder (2 g, 35.6 mmol) and H_2O (25 mL) were poured in sequence. The mixture was left stirring at 100°C for 3 hours. After this time, the reaction mixture was cooled down to room temperature and removed from the flask using deionized water. The resulting suspension was poured in a Büchner funnel and washed with water under vacuum up to neutral pH. The obtained solid was put in a stove for 6 hours to remove excess water. 6.5 g of black powder were obtained. ATR-FTIR ν_{max} 3390 (O–H stretch, broad), 1560 (C=C stretch), 1140 (C–O stretch), 983 (O–H bend), 850 (out of plane, =C–H bend, monosubst) cm^{-1}.

S2. Characterization of HSAG, G-OH and PU

Boehm titration

Boehm titration was performed to quantitatively determine the content of oxygenated surface groups. 100 mg of G-OH were poured in a becker with 50 ml of NaOH 0.0492 M. The mixture was left to stir at room temperature for 24 hours. After this time, the suspension was filtrated. CO_2 was removed from solution immediately before the titration: samples were poured in 40 mL glass vials equipped with a glass septum lids. N_2 was bubbled into the vial through a needle submerged in the solution. Bubbling rate was less than 1 mL/min. The time of degasification was 24 h. After degasification, the samples were transferred to a beaker that had been purged with the inert gas and covered with Parafilm®, to prevent absorption of atmospheric CO_2. 10 ml of the filtered product was withdrawn with a pipette and placed in a becker with the addition of 20 ml of HCl 0.05 M and 2 drops of phenolphthalein. The solution was titrated with NaOH 0.0492 M. The concentration of OH groups was calculated by applying Eq S1.

$$Xc = \frac{M_{NaOH}\left(y_{ml} + \frac{V_{NaOH}}{5}\right) - M_{HCl}(V_{HCl})}{\frac{m_{G-OH}}{5}} \quad (S1)$$

where:

M_{NaOH} is the molar concentration of the initial solution; y_{ml} is the volume of NaOH taken from the suspension after filtration of the allotrope; V_{NaOH} is the volume of NaOH; M_{HCl} is the molar concentration of HCl; V_{HCl} is the volume added to the filtered product; m_{G-OH} are the grams of G-OH.

Infrared spectroscopy

FTIR absorption spectra were recorded by using a Thermo Scientific spectrometer in transmission mode using a diamond anvil cell (DAC) coupled with a ThermoElectron FTIR Continuµm IR microscope (resolution: 4 cm^{-1}; scans: 128; absorption from 400 to 4000 cm^{-1}).

Raman spectroscopy

Raman spectra of powder samples were recorded with a Horiba Jobin Yvon Labram HR800 dispersive Raman spectrometer equipped with an Olympus BX41 microscope and a 50X objective (resolution: 2 cm^{-1}; acquisition time: 30 seconds and 4 accumulation). The excitation line at 514.5 nm of an Ar^+ laser was kept at 0.5 mW in order to prevent possible photo induced thermal degradation of the samples. Each Raman spectrum reported was obtained as average of four spectra recorded in different points of the sample.

Wide angle X-ray diffraction

WAXD patterns were obtained in reflection, with an automatic Bruker D8 Advance diffractometer, with nickel filtered Cu–K_α radiation. Patterns were recorded in 4° – 80° as the 2θ range, being 2θ the peak diffraction angle. Distance between crystallographic planes of HSAG was calculated from the Bragg law. The $D_{hk\ell}$ correlation length, in the direction perpendicular to the *hkl* crystal graphitic planes, was determined applying the Scherrer equation:

$$D_{hk\ell} = K\,\lambda\,/\,(\beta_{hk\ell}\cos\theta_{hk\ell}) \quad (S2)$$

where K is the Scherrer constant, λ is the wavelength of the irradiating beam (1.5419 Å, Cu-K_α), $\beta_{hk\ell}$ is the width at half height, and $\theta_{hk\ell}$ is the diffraction angle. The instrumental broadening, *b*, was determined by obtaining a WAXD pattern of a standard silicon powder 325 mesh (99%), under the same experimental conditions. The width at half height $\beta_{hk\ell} = (B_{hk\ell} - b)$ was corrected, for each observed reflection with $\beta_{hk\ell} < 1°$, by subtracting the instrumental broadening of the closest silicon reflection from the experimental width at half height, $B_{hk\ell}$.

Thermogravimetric analysis (TGA)

TGA tests under flowing N_2 (60 mL/min) were performed with a Mettler TGA SDTA/851 instrument according to the standard method ISO9924-1. Samples (10 mg) were heated from 30 to 300°C at 10°C/min, kept at 300°C for 10 min, and then heated up to 550°C at 20°C/min. After being maintained at 550°C for 15 min, they were further heated up to 700°C and kept at 700°C for 30 min under flowing air (60 mL/min).

Differential scanning calorimetry (DSC)

DSC analyses under N_2 (80 mL/min) atmosphere were performed with a Mettler DSC 823 calorimeter. Each sample (4.5 mg ± 0.05 mg) was kept at 50°C for 5 min, cooled to -85°C at 10°C/min, kept 10 min at this temperature, heated up to 50°C at 5°C/min.

S3. FT-IR investigation on sp^2 carbon allotropes

The spectrum of HSAG (Figure S1 A) is characterized by the feature centered at 1590 cm^{-1} which is the absorption peak of graphite and graphene materials, assigned to E_{1u} IR active mode of collective C=C stretching vibration. In the spectra of G-OH (Figure S1 B), this peak (centered at 1590 cm^{-1}) is present along with new bands. These new bands can be assigned to the absorption of different –OH groups bonded to the graphene sheets. The corresponding peaks are located at: i) 3400 cm^{-1}, assigned to the –OH stretching vibrations of hydrogen bonded hydroxyl groups (broad absorption); ii) 1388 cm^{-1}, assigned to the out of plane vibration of -OH groups; iii) 1110 cm^{-1}, assigned to C-O stretching vibration and iv) 970 cm^{-1}, assigned to the in plane phenyl-O-H bending.

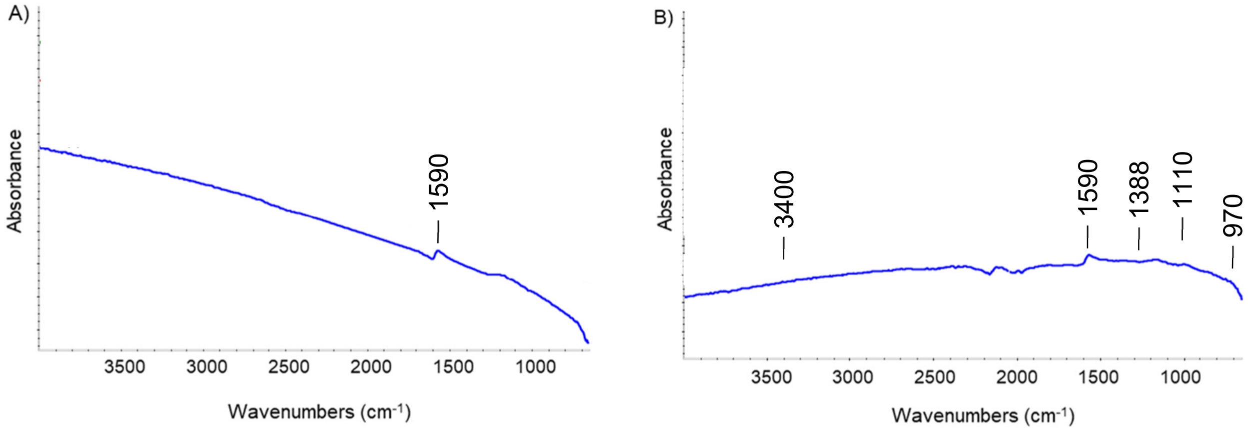

Figure S1. (A) FT-IR spectra of HSAG; (B) FT-IR spectra of G-OH.

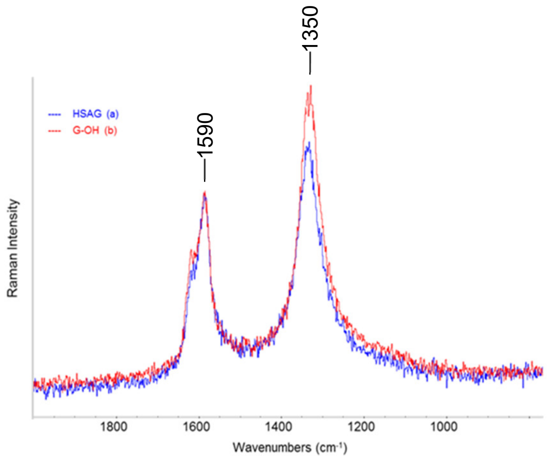

Figure S2. Raman spectra with normalized intensities of HSAG (a), G-OH (b).

S4. Calculation of the Hansen Solubility Parameters and Hansen Solubility Sphere

The following equation describes the interaction between a solvent and a solute:

$$\Delta G_{\mathrm{M}} = \Delta H_{\mathrm{M}} - T\Delta S_{\mathrm{M}}, \qquad \text{(S3)}$$

where ΔG_M is the Gibbs free energy change of mixing, ΔH_M is the enthalpy change of mixing, T is the absolute temperature, and ΔS_M is the entropy change of mixing.

To have a spontaneous mixing, the free energy change should have a negative value: $\Delta G_M < 0$. In the case of a small positive entropy change for the dissolution of a solute, the sign of ΔG_M is prevailingly determined by ΔH_{M}.

Hildebrand reported that the solubility of a substance, in a series of solvents, is due to the internal pressures of the solvents. The Hildebrand theory was developed by Scatchard, who proposed the concept of cohesive energy density. Then, Hildebrand and Scott [1] introduced the following equation to describe the enthalpy of mixing:

$$\Delta H_M = V_M[(\Delta E_1{}^V/V_1)^{1/2} - (\Delta E_2{}^V/V_2)^{1/2}]^2\phi_1\phi_2, \qquad \text{(S4)}$$

where V_M is the volume of the mixture; $\Delta E_i{}^V$ is the energy of vaporization of species i, i.e. the change of energy for the isothermal vaporization of a saturated liquid to an ideal gas state of infinite volume; V_i is the molar volume of species i, and ϕ_i is the volume fraction of species i in the mixture.

The cohesive energy E of a material is defined as the difference (increase) of the internal energy per mole of a material upon removing all of the intermolecular forces. The cohesive energy density (CED) of a material is the energy needed to overcome all the intermolecular forces per unit volume and is expressed by the following equation:

$$CED = E/V = (\Delta H_{VAP} - RT)/RT, \qquad \text{(S5)}$$

where ΔH_{VAP} is the enthalpy of vaporization.

The following equation defines the Hildebrand solubility parameter as the square root of the cohesive energy density:

$$\delta = (E/V)^{1/2} \qquad \text{(S6)}$$

By combining Equation (S6) with Equation (S4), the following equation is obtained, to express the enthalpy of mixing per unit volume, for a binary mixture:

$$\Delta H_M/V = (\delta_1 - \delta_2)^2\phi_1\phi_2, \qquad \text{(S7)}$$

According to Equation (S7), to have spontaneous mixing, the entropy of mixing should be higher than the enthalpy of mixing.

It can be thus concluded that, to have miscibility over the whole range of volume fractions, the difference in solubility parameters should be small, if any.

Specific interactions between molecules, such as hydrogen bonding, are not considered by the Hildebrand method. Hansen developed a method that defines the solubility parameters on the basis of three specific interactions. In fact, the cohesive energy E is given by three contributions, which are due to dispersion (*D*), polar–polar (*P*), and hydrogen bonding (*H*) forces:

$$E = E_{\mathrm{D}} + E_{\mathrm{P}} + E_{\mathrm{H}}. \qquad \text{(S8)}$$

By dividing all the terms in Equation (S8) by the molar volume, one obtains:

$$E/V = E_{\mathrm{D}}/V + E_{\mathrm{P}}/V + E_{\mathrm{H}}/V. \qquad \text{(S9)}$$

As suggested by Equation (S9), the square of the total, Hildebrand, solubility parameter is the sum of the squares of the Hansen components:

$$\delta_T{}^2 = \delta_D{}^2 + \delta_P{}^2 + \delta_H{}^2. \qquad \text{(S10)}$$

A Hansen parameters space can be defined. In this space, a solute is identified by three coordinates (δ_D, δ_P and δ_H), which are the Hansen solubility parameters (HSP). The

difference between the cohesive energy density of two substances (a solute and a solvent) correlates with the distance between two points in the Hansen space. The cohesive energy density difference correlates as well with the enthalpy of mixing, which is low (minimal) when two substances are miscible. Hence, two points close to each other in the Hansen space correspond to miscible compounds.

The HSP of a solute *i* is estimated by means of a dispersion test carried out on different solvents *j*,: good solvents give stable solutions/dispersions, which are not obtained with bad solvents. By using the parameters (coordinates) of the solvents, a sphere is defined, centered on the solutes solubility parameters, which includes the good solvents and excludes the non-solvents. The sphere radius is defined as R_0, named as radius of interaction, and the distance between the solute and the solvent is R_a, given by the following equation:

$$R_{a,ij}{}^2 = 4(\delta_{D,i} - \delta_{D,j})^2 + (\delta_{P,i} - \delta_{P,j})^2 + (\delta_{H,i} - \delta_{H,j})^2 \quad \text{(S11)}$$

Equation (S12) defines the ratio between $R_{a,ij}$ and R_0 as RED, relative energy difference. When solutes and solvents have good affinity, RED is < 1.

$$RED = \frac{R_{a,ij}}{R_o} \quad \text{(S4)}$$

The coordinates of the center of the Hansen solubility sphere are calculated by minimizing the radius of interaction (the distance from the coordinates of the good solvents), excluding the bad solvents (RED > 1) and including the good ones (RED < 1). The three unknown HSP of the solute are given by the coordinates of the sphere center.

Such a procedure was performed by using a MATLAB algorithm that is shown in Figure S3.

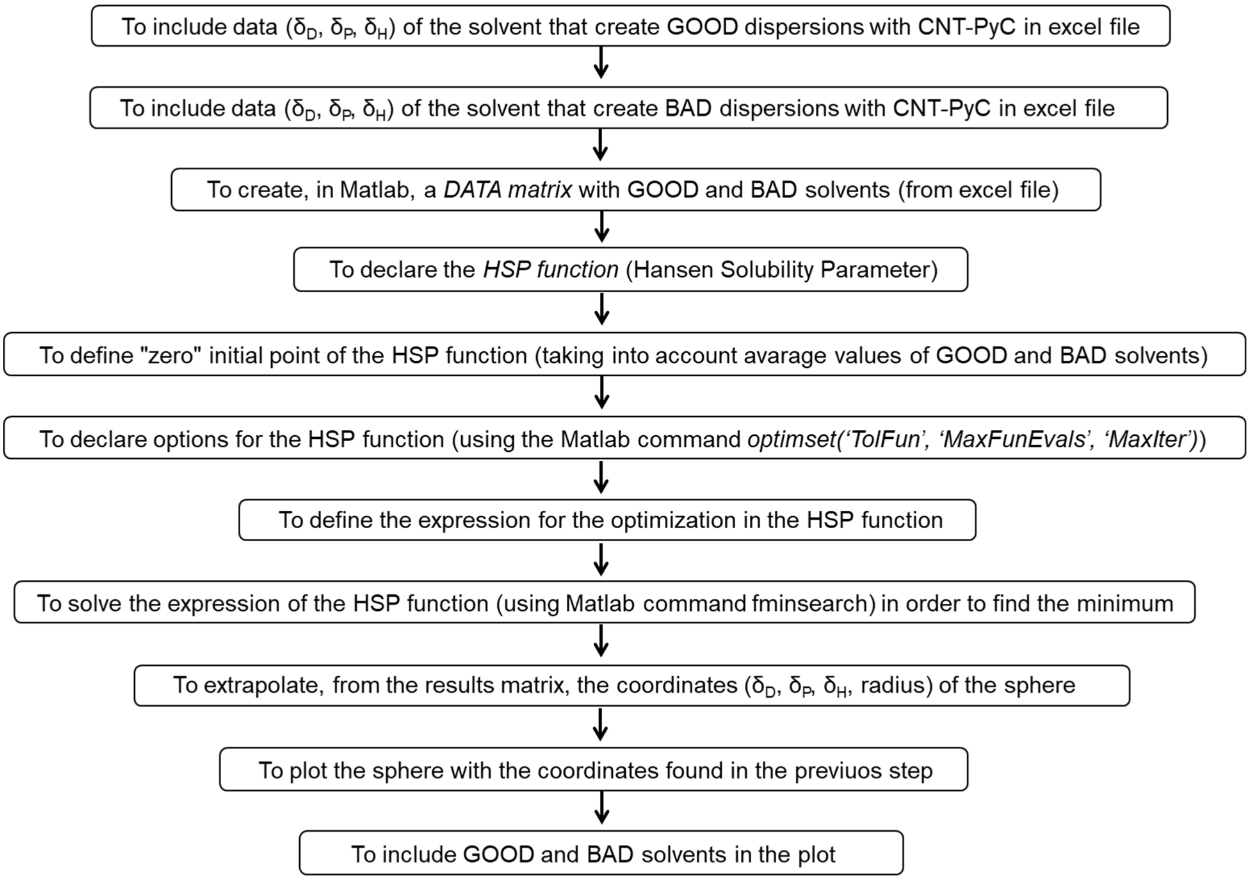

Figure S3. MATLAB algorithm used for the preparation of Hansen's sphere.

Table S1. Hansen solubility parameters for selected solvents and results of inspection of dispersions of GOH adducts after 1 week[a,b.]

**Solvent**	δ_D **[MPa$^{1/2}$]**	δ_P **[MPa$^{1/2}$]**	δ_H **[MPa$^{1/2}$]**	**Affinity**	
				HSAG	G-OH
**water**	**18.1**	**17.1**	16.9	bad	good
methanol	15.1	12.3	22.3	bad	good
glycol	16.8	9.4	23.3	good	good
2-propanol	15.8	6.1	16.4	bad	good
acetone	15.5	10.4	7	bad	good
ethyl acetate	15.8	5.3	7.2	bad	good
chloroform	17.8	3.1	5.7	good	good
xylene	17.6	1	3.1	bad	bad
toluene	18	1.4	2	bad	bad
hexane	14.9	0	0	bad	bad

[a] concentration: 1mg/mL; [b]good: homogenous dispersion was observed soon after sonication and after one week storage at rest; bad: separation of adduct from the solvent

S5. Polyol dispersions of G-OH

Polyol dispersions of G and G-OH adducts were prepared with different concentrations (1, 0.1, 0.05, 0.01, 0.005 and 0.001 mg/mL) as described in the experimental part. Figure S4 shows dispersions of G-OH (1 mg/mL) at rest, after 1 month storage, and in a range of concentration from 0.1, to 0.001 mg/mL.

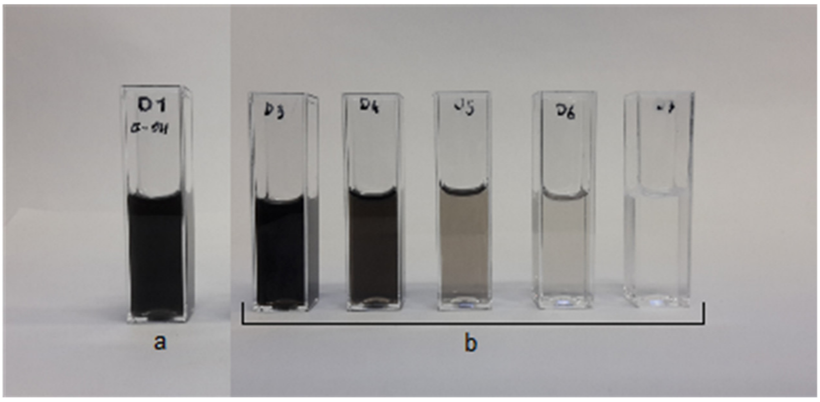

Figure S4. Polyol suspensions of G-OH (1 mg/mL) at rest (a) and at concentration of 0.1, 0.05, 0.01, 0.005 and 0.001 mg/mL (b) (from right to left).

Figure S5 reports results from UV-Vis absorption analysis. Figure S5A shows the absorbance detected for the freshly prepared G-OH polyol suspensions (1 mg/mL). Figure S5B shows that the absorbance monotonously increases with G-OH concentration.

A)

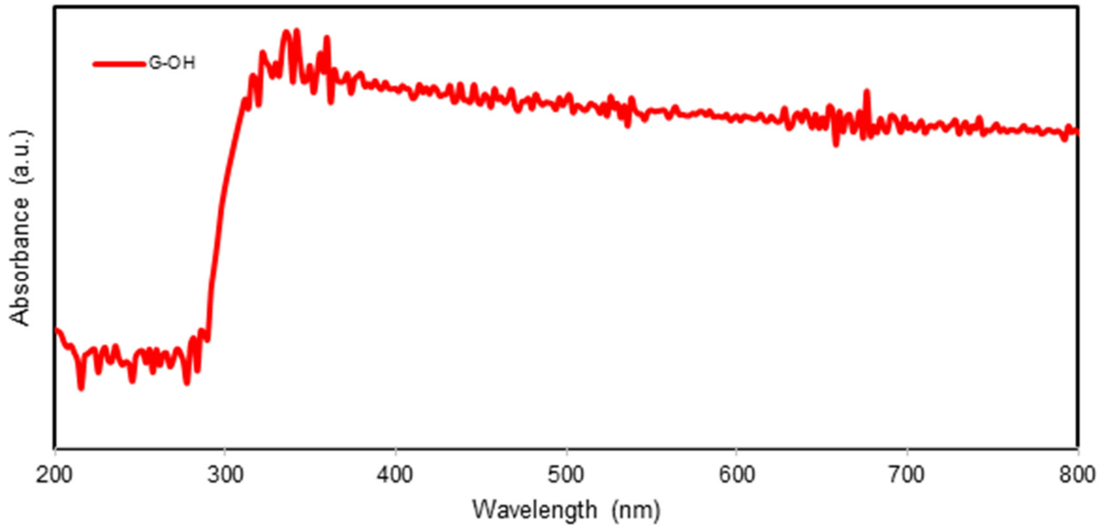

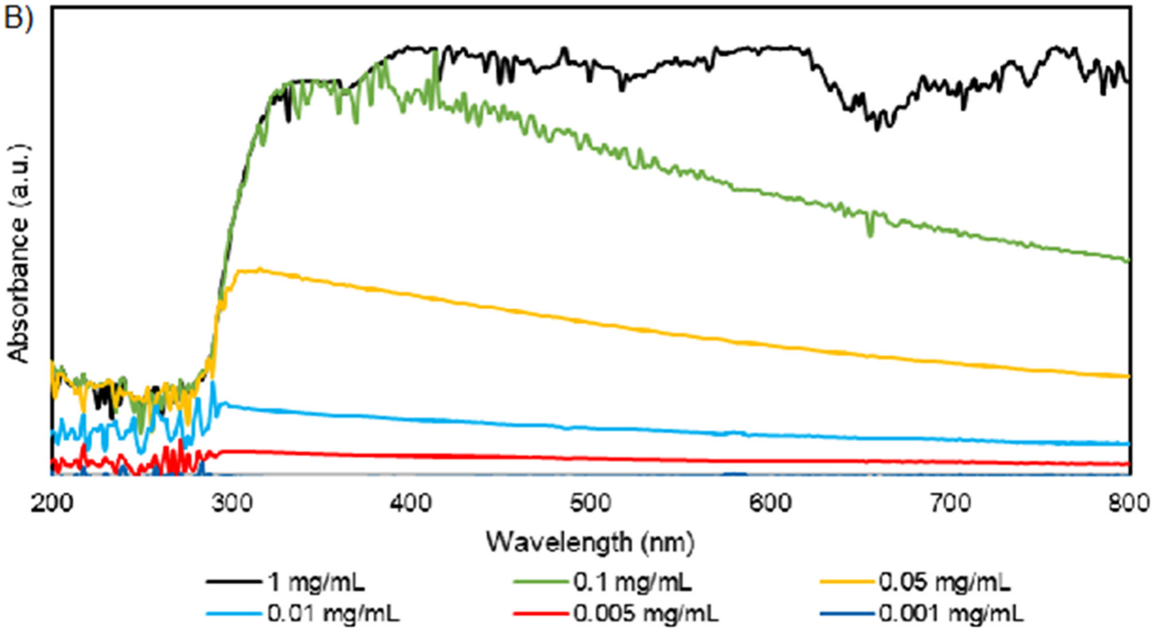

Figure S5. (A) UV-Vis traces of polyol dispersions of G-OH. (B) G-OH dispersions at different concentrations (1, 0.1, 0.05, 0.01, 0.005, 0.001 mg/mL).

S6. WAXD

In pristine HSAG, crystalline order in the direction orthogonal to structural layers is revealed by two (00ℓ) reflections: 002 at 26.6°, that corresponds to an interlayer distance of 0.338 nm, and 004 at 54.3°. Such interlayer distance is slightly larger than the one of ordered graphite samples (d_{002} = 0.335 nm). The in plane order is shown by 100 and 110 reflections, at 42.5° and 77.6° respectively. By applying the Scherrer equation (see the experimental section) to (002) and (110) reflections, respectively, the out of plane ($D_{\perp}$) and the in plane ($D_{\parallel}$) correlation lengths were calculated resulting as 9.8 nm for ($D_{\perp}$) and 30.2 nm for ($D_{\parallel}$). The in-plane correlation length is thus larger than the out-of-plane correlation length. From the values of ($D_{\perp}$) and of the interlayer distance, the number of stacked layers was estimated to be about 35. These results reveal the turbostratic nature of HSAG, that has however remarkable crystalline order inside the structural layers. (002) reflection in the patterns of G-OH samples remains at the same 2θ value, indicating that the oxidation reaction did not promote expansion of the interlayer distance. The number of stacked layers in G-OH sample was calculated by applying the Scherrer equation to 002 reflection and was found to be 25.

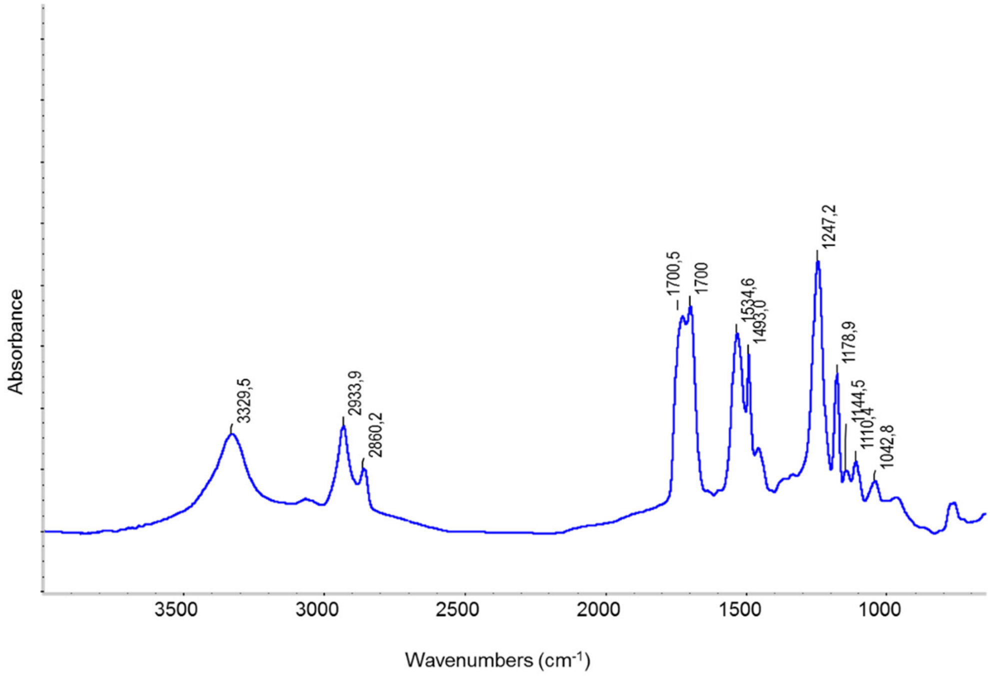

Figure S6. FT-IR spectrum of PU from 1,2-catechol, 1,4-butanediol and hexamethylene diisocyanate.

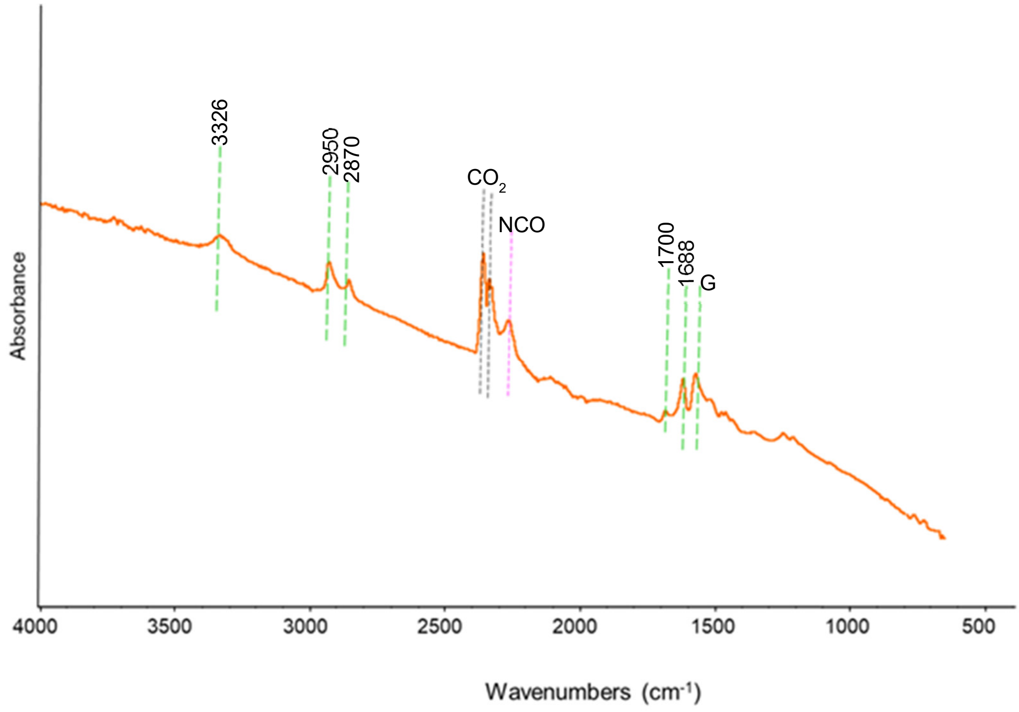

Figure S7. FT-IR spectrum of the reaction of 1-butanol with butylisocyanate and G-OH.
